#  Brucella pericarditis: A forgotten cause of chest pain 

**DOI:** 10.22088/cjim.8.2.116

**Published:** 2017

**Authors:** Feridoun Sabzi, Reza Faraji

**Affiliations:** 1 Preventive Cardiovascular Research Centre, Kermanshah University of Medical Sciences, Kermanshah, Iran.

**Keywords:** Chest pain, *Brucella*, Cardiovascular system

## Abstract

**Background:**

Brucellosis pericarditis is an extremely rare involvement of the cardiovascular system with *Brucella* species that has worldwide distribution and is endemic in many provinces of Iran.

**Case presentation:**

The present report describes an exceptional case of pericardial involvement by *Brucella melitensis* in a 50- year-old woman. The patient presented with night sweats, mild fever, weight loss, and chest pain.

**Conclusion:**

Pericarditis occurs during the course of brucellosis as an isolated cardiac symptom and may be asymptomatic or may be manifested with chest pain as ischemic heart disease.

Humans are likely to become infected with brucellosis through consumption of raw or nonpasteurized milk or soft cheese made of fresh milk (1). The incidence of cardiovascular involvement in Brucellosis, such as endocarditis, myocarditis or pericarditis was reported to be as low of 1% of cases and isolated pericarditis is exceedingly less frequent than concomitant with endocarditis or myocarditis (2). Andriopoulos found that pericarditis or pericardial effusion in brucellosis is thought to develop almost exclusively in the presence of endocarditis (3). Our case was unique because the patient presented with severe chest pain and isolated pericardial effusion and in the absence of concomitant myocarditis or endocarditis.

## Case presentation

A 50-year-old woman was admitted in a general hospital with severe shooting chest pain, malaise, fatigue, and low-grade fever. He worked and lived in rural villages and consumed fresh cheese, milk and other animal raw unpasteurized products. On physical examination, stethoscope revealed a friction rub of the pericardium on anterior chest wall. The laboratory exam revealed anemia 10.5 g/DL and hepatic function tests were normal. Electrocardiography (ECG) with sinus rhythm revealed an ST-segment change in pericardial leads. Chest x-ray revealed the upper normal cardiac silhouette. Stool, urine, and pharyngeal cultures, and investigations for Epstein-Barr virus, (**Epstein-Barr virus VCA antibody (IgM** <20U/mL) cytomegalovirus (negative: IgM), adenovirus, (adenovirus antibody, IgG < 1:10, adenovirus antibody, IgM <1:10), *Chlamydia pneumoniae* (IgM <20 U/ml) and *Mycoplasma pneumonia* (IgG <160 U/m), were negative. Mycobacteria culture, mycobacteria via PCR, and Ziehl-Neelsen staining for ARB yielded negative results. The patient’s PPD test was also negative (7 mm) and enzyme-linked immunosorbent assay tests for anti-*Brucella* IgG and IgM antibodies were also strongly positive (133 U/ml and 31.7 U/ml; 30 U/ml and 20 U/ml), respectively.

Wright test titer, Coombs wright and 2 mercaptoethanol (2ME) were 1/5120, 1/1280, 1/640 respectively. In addition, *Brucella melitensis* were isolated from two of three consecutive blood cultures. The patient remained symptomatic with chest pain but with no signs of heart failure or arrhythmias on serial ECG during the hospital stay. 

**Figure 1:**
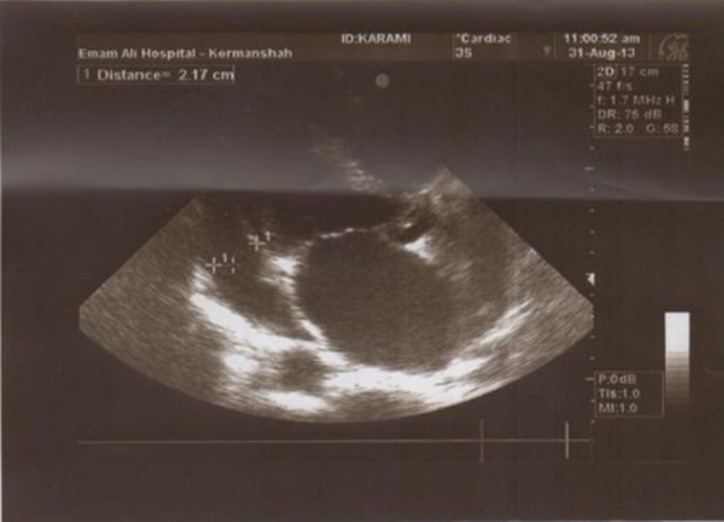
Echocardiography in four chamber view shows pericardial effusion

**Figure 2:**
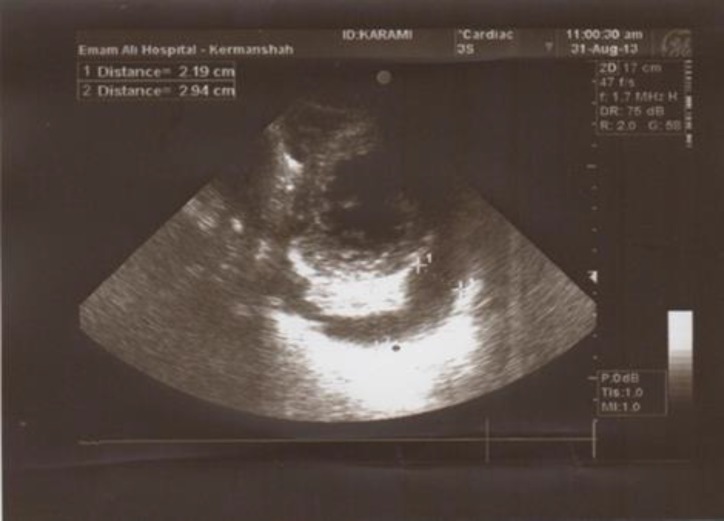
Apical four chamber view shows pericardial effusion

The creatine kinase was normal from the normal level on admission over the following hospital days, as were the levels of the other enzymes. Also, transesophageal echocardiogram noted normal aortic valve and absence of root abscess and showed the normal mitral valve. In echocardiography there was also a mild pericardial effusion without signs of cardiac tamponade with pericardial thickening and fibrins intraperiodical materials (figures 1, 2). 

Additional laboratory blood tests were carried out because of pericardial effusion, including tumor markers, rheumatoid factor, antinuclear antibodies, anti-double-stranded DNA antibodies, and serum immunoglobulin but did not reveal the cause. However, C-reactive protein (CRP), ESR and fibrinogen were strongly positive. 

Angiography revealed normal coronary artery and ruled out coronary artery disease as the cause of chest pain. Our patient was given oral ibuprofen 400mg three times daily and doxycycline 100mg twice daily plus oral rifampicin 900mg once daily, with intramuscular streptomycin 1 g once daily for the first three weeks. His cardiac chest pain symptoms regressed rapidly 10 days after the initiation of anti-inflammatory drug and antibiotic treatment. The treatment continued for three more weeks and no recurrence was observed during the six-month follow-up. 

## Discussion

Pappas et al. (4) reported that in cardiovascular involvement by brucellosis, the simultaneous onset of myocarditis and pericarditis together is common, but the isolated appearance of pericarditis is extremely rare. In Ataman et al.’s (5) studies after carrying out a search on medical literature database using the words “pericarditis” and “brucellosis” 18 references were identified during a 30-year period. Of these, only five documented papers contained reports of infections in humans with pericarditis associated with brucellosis, Anguita et al. (6) reported two cases of *Brucella melitensis* pericarditis. Neither of these two patients had chest pain. Delmastro et al. (7) reported a case of Brucellosis pericarditis with shooting chest pain in a patient with acute Brucellosis. Gomez-Huelgas et al. (8) reported a case of pericarditis with acute signs and symptoms and pericardial effusion without chest pain. Anguita reported that isolated pericardial or myocardial involvement is exceptional, even less so in association with brucellosis. In a case series reported by Ataman et al. when the clinical manifestations were evaluated in 240 subjects with brucellosis, signs and symptoms suggestive of pericarditis were found in 0.8% (5). 

Also in another study performed in Turkey on 1028 cases of brucellosis that were analyzed, pericardial involvement was detected in 0.7% of cases (9). In a study reported by Ugartemendía et al., isolated pericarditis and massive pericardial effusion were the important clinical findings that progressed to cardiac tamponade (10). 

This study had an important difference with previous papers, where the subject with pericarditis was asymptomatic and the significant clinical signs and symptoms were fever and poor general condition. In a case similar to ours reported by Gatselis et al., a massive pericardial effusion was detected at the Trus thoracic echocardiography (TEE), which was performed to evaluate endocarditis, because while the case had a systolic souffle on auscultation of pericardium, he was asymptomatic (11).

 The probability of infective endocarditis was excluded in this case due to the absence of variables such as vegetations on TTE, the absence of a new cardiac souffle, and recovery of chest pain after starting anti-brucella drugs therapy. Although *Brucella* pericarditis is very unusual, it should always be considered in a country where brucellosis is endemic (9).

In conclusion,** c**ardiac involvement is not common in human brucellosis, with the most common manifestation being aortic endocarditis. Pericarditis occurs during the course of brucellosis as an isolated cardiac symptom and may be asymptomatic or may be manifested with chest pain as ischemic heart disease. 

This case shows that in endemic areas, *Brucella* can be considered as a potential causative agent of severe chest pain and pericardial effusion, even in the absence of concomitant endocarditis. This possibility could be considered particularly in cases where infection of brucellosis is possible. 
